# Anxiety among healthcare professionals during the COVID-19 pandemic: Predictive role of social support

**DOI:** 10.1192/j.eurpsy.2021.1785

**Published:** 2021-08-13

**Authors:** A. Bouaziz, N. Smaoui, M. Kraiem, M. Maalej Bouali, S. Omri, R. Feki, J. Ben Thabet, L. Zouari, N. Charfi, M. Maalej

**Affiliations:** 1 Psychiatry C Departement, Hospital University of HEDI CHAKER, sfax, Tunisia; 2 Psychiatry C Department, Hedi chaker University hospital, sfax, Tunisia; 3 Psychiatry C, Hedi Chaker University Hospital, sfax, Tunisia

**Keywords:** Healthcare professionals, Trait-Anxiety, Social support, Covid-19 pandemic

## Abstract

**Introduction:**

The COVID-19 pandemic may cause elevated levels of anxiety in healthcare professionals (HCP). Identifying factors that could help maintain mental health especially social support could be helpful in facing this stressful situation.

**Objectives:**

The aim of this study was to asses the relationship between the trait-anxiety and perceived social support among Tunisian HCP in the current pandemic wave of COVID-19.

**Methods:**

A cross- sectional descriptive and analytic study conducted among Tunisian HCP during November and December 2020. The data were collected by an online questionnaire. The trait-anxiety was assessed using the “General Anxiety questionnaire of Spielberger” (STAI-Y-B). We used the “Social Support Questionnaire” to measure availability and satisfaction regarding perceived social support.

**Results:**

Participants were 135 HCP, and aged from 24 to 61 years old (average age 31.98 years). The sex ratio was 1.1 (71 males and 64 females). Of HCP involved in the study, 61.5% were single, 36.3% were married and 2.2% were divorced. The average scores of availability and satisfaction regarding perceived social were 7.79 (SD=3.56) and 28.41 (SD=6.75), respectively. Seventy-two of the HCP had a trait-anxious. Analysis showed that social support satisfaction scores were significantly lower in trait-anxious HCP (p<0.001). However, there was no significant difference in the score of availability according to trait-anxiety (p=0.49).

**Conclusions:**

Our study highlighted the existence of a deficit on perceived social support satisfaction among trait-anxious Tunisian HCP. Perceived social support as a determinant of trait anxiety should be the focus of social work in this period.

**Disclosure:**

No significant relationships.

